# Validated HPLC Method for Concurrent Determination of Antipyrine, Carbamazepine, Furosemide and Phenytoin and its Application in Assessment of Drug Permeability through Caco-2 Cell Monolayers

**DOI:** 10.3797/scipharm.1109-03

**Published:** 2011-10-22

**Authors:** Sachin Ramrao Patil, Lokesh Kumar, Gunjan Kohli, Arvind Kumar Bansal

**Affiliations:** 1 Department of Pharmaceutical Technology (Formulations), National Institute of Pharmaceutical Education and Research (NIPER), S.A.S. Nagar, Mohali, Punjab-160 062, India; 2 Department of Pharmaceutics, National Institute of Pharmaceutical Education and Research (NIPER), S.A.S. Nagar, Mohali, Punjab-160 062, India

**Keywords:** Validation, HPLC, Caco-2 cell monolayer, Phenazone, Carbamazepine, Furosemide, Phenytoin

## Abstract

The present work explains the development and validation of a simple, rapid and sensitive liquid chromatographic method for the simultaneous determination of antipyrine (ANT), carbamazepine (CBZ), furosemide (FSD) and phenytoin (PHTN). Chromatographic analysis was carried out by a reversed phase technique on a C18 column, using water pH 3.0 and 50:50 mixtures of methanol and acetonitrile (58:42 v/v) as the mobile phase, at a flow-rate of 1.0 ml/min and a column temperature of 40°C. Detection was carried out at 205 nm for CBZ and PHTN and at 230 nm for ANT and FSD. The proposed method was evaluated for validation parameters including linearity, range, accuracy, precision, limit of detection (LOD), limit of quantification (LOQ) and specificity. Elution of drugs ANT, FSD, PHTN, and CBZ was observed at 4.1, 5.1, 12.3 and 13.5 min, respectively. The method was found to be linear (R^2^ ≥ 0.999) in the concentration range of 5–100 μM, with an acceptable accuracy and relative standard deviation. Results of intra- and inter-day validation (n=3) showed the method to be efficient for routine determination of these permeability markers in Caco-2 cell monolayer permeability studies. The method was successfully utilized for determination of standard compounds in Caco-2 permeability experiments.

## Introduction

The Caco-2 cell monolayer is widely used for the determination of drug intestinal permeability and/or absorption [1–3]. According to US FDA guidance, rank order relationship between the Caco-2 permeability data and *in-vivo* human absorption data for at least 20 model drugs need to be established to demonstrate the method suitability of Caco-2 cell monolayer permeability model [4]. These drugs are selected from the class of high as well as low permeability drugs to demonstrate the utility of the method for both high as well as low permeability drugs. The present work deals with the development of an analytical method for simultaneous routine determination of high permeability markers – antipyrine (ANT), carbamazepine (CBZ), and phenytoin (PHTN) – and low permeability marker – furosemide (FSD). These four compounds act as internal permeability standards for routinely monitoring of the inter-day variability of Caco-2 cell monolayer model, as well as for testing the integrity and functioning of Caco-2 cell monolayer. Physicochemical properties of the four molecules are reported in Table 1. Individual analytical method for HPLC determination of antipyrine [5–7], carbamazepine [8–11], furosemide [12–15] and phenytoin [16–19] have already been reported. However, analysis by a single analytical method would simplify their use in cocktail approach as standard permeability markers and also avoid validation of analytical method for each drug [20]. The reported method can also be modified to suit other models for permeability study like MDCK cells or intestinal perfusion models.

## Materials and Methods

### Materials

ANT and PHTN were purchased from Sigma (St. Louis, MO, USA). CBZ and FSD were a gift from Cadila Pharmaceuticals and IPCA laboratories Ltd. (India), respectively. HPLC grade acetonitrile and methanol were obtained from Mallinckrodt Baker Inc. (USA) and Ultrapure^®^ water from ELGA (Bucks, UK) was used for analysis. *O*-phosphoric acid and DMSO were from Sigma (USA). All other chemicals and reagents were of analytical or HPLC grade as appropriate.

Caco-2 cells (HTB-37) were purchased from American Type Culture Collection (ATCC), Manassas, VA at a passage no. 18. Cells were cultured in T-75 cm^2^ tissue culture flasks obtained from Cellstar^®^, Greiner Bio-One (Germany). Hanks balanced salt solution (HBSS) and Dulbecco’s modified eagle’s medium (DMEM) containing 4.5 g/L glucose, L-glutamine, D-glucose, sodium pyruvate was obtained from Sigma, USA. Non essential amino acid solution (NEAA), fetal bovine serum (FBS) and trypsin-ethylenediamine tetraacetic acid solution were purchased from GIBCO, Invitrogen, USA. Penicillin-streptomycin-amphotericin solution, phosphate buffered saline (PBS) and 2-[4-(2-hydroxyethyl)-1-piperazinyl]ethanesulphonic acid (HEPES) were from HiMedia, India. Milli-Q grade water purified by a Milli-Q UV Purification System (Millipore, Bedford, MA, USA) was used.

### Cell culture

Caco-2 cells were cultured in T-75 cm^2^ tissue culture flask and maintained by sub-culturing at a split ratio of 1:4 to 1:6 when reached to 80–90% confluency. The culture medium contained DMEM with 1% NEAA, 1% Penicillin-streptomycin-amphotericin solution and 15% FBS. The cells were grown in an atmosphere of 5% CO_2_ at 37°C and 95% humidity with replacement of culture medium after 2–3 days.

For permeability studies, Caco-2 cells were seeded on a 24-well plate at a seeding density of 75000 cells/insert, in 0.4 μm pore size polycarbonate tissue culture inserts (Millicell^®^, cell culture plate assembly, Millipore, Bedford, USA). Caco-2 cells were used at a passage of 34–40 for transport studies at 21–25 days after seeding, and the culture medium in the inserts (400 μl on apical side and 800 μl on basolateral side) was replaced every alternate day. The trans-epithelial electric resistance (TEER), expressed in Ohm.cm^2^, was measured using a Millicell-ERS voltohmmeter (Millipore, Bedford, USA). Paracellular transport marker lucifer yellow was used to confirm the integrity of Caco-2 monolayers. Lucifer yellow was quantified at excitation and emission wavelengths of 485 nm and 530 nm, respectively, with a spectrofluorimeter (Shimadzu, Japan). The monolayer used for the transport experiments had TEER values greater than 300 Ω.cm^2^ and the transport rate of lucifer yellow was less than 1% per hour.

### Transport studies

After Caco-2 cells were grown on filters in the 24-well plate for 21–26 days, the cell monolayer was washed twice with PBS pH 7.4 to remove the traces of DMEM. After washing, the plates were incubated with transport buffer for 30 min at 37°C in CO_2_ incubator and trans-epithelial electrical resistance (TEER) of the monolayer was measured. TEER value was corrected by subtracting the TEER of blank inserts from TEER of monolayer. Transport buffer was then removed gently by aspiration. For apical to basolateral transport study (A→B), 400 μl of drug solution (ANT, CBZ, FSD and PHTN) in transport buffer was added to apical side [10] and 800 μl of blank transport buffer was added to the basolateral side (BL). For basolateral to apical transport study (B→A), the drug solution was placed on the basolateral side and blank transport buffer on the other side. Samples of 300 μl were withdrawn from respective compartments at 15, 30, 45, 60, 90, and 120 min and the volume withdrawn was replaced with blank transport buffer each time. Experiment was performed in shaker incubator at 37°C and 75 rpm. The samples collected from each time point were stored at −20°C, until determined by HPLC with PDA detector.

### Data Analysis

Data from three independent experiments are presented as mean±S.D. Results of bidirectional transport are expressed as permeability coefficient (nm/s), which was calculated using the equation:

$${\text{P}}_{\text{app}}=\frac{\text{dQ}/\text{dt}}{\text{A}\times {\text{C}}_{0}}$$

Where,

dQ/dt is the slope of the cumulative drug transported vs time curve (μg/sec)

A is the surface area of the filter (cm^2^)

C_0_ is the initial concentration of the drug (μM)

P_app_ is the apparent permeability (cm/sec)

### Equipments/Chromatographic system

Chromatographic measurements were performed on a Shimadzu HPLC system (Shimadzu, Kyoto, Japan) equipped with a model series SPD-M20A photodiode array detector, a gradient elution pump with degassing device DGU-20A5, a cooling auto-sampler SIL-20AC, a column heater/cooler CTO-10A VP and a system controller CBM-20A. The diode array detector was used for the spectrum extraction, while the analysis was carried out at 205 nm and 230 nm. Separations were performed at 35°C using a C18 (250 mm × 4.6 mm, 5 μm) stationary phase. Data was acquired via Class VP data acquisition software, version 6.12 SP1. AG 285 Mettler-Toledo^®^ balance (Switzerland) was used for weighing standards. In addition, Millipore filters and pH meter Cyberscan 510 Eutech Instruments Ltd. (Singapore) were used in the study. The mobile phase solvents were filtered through Millipore™ (nylon) 0.45 μm filters before use in HPLC.

### HPLC Parameters

The chromatographic separation was carried out using a mobile phase consisting of Ultrapure^®^ water (to pH 3.0 with 20% ortho-phosphoric acid) and a mixture of acetonitrile and methanol in 50:50 ratio at an isocratic mode with 58:42 proportions. The injection volume was 10 μl, and the mobile-phase flow rate was set to 1 ml min ^−1^. Detection was carried out at 205 nm for CBZ and PHTN and at 230 nm for ANT and FSD.

### Standard and sample preparation

Primary stock solution of all drugs, i.e., ANT, CBZ, FSD, and PHTN was prepared in methanol to obtain a concentration of 10 mM. Consequently, the primary stock solution was diluted with HBSS buffer to prepare a secondary stock solution of 100 μM concentration. Secondary stock solution was diluted with HBSS to achieve concentrations in the range of 5–100 μM.

### Validation Studies

The proposed HPLC method was validated for various parameters, viz. linearity, range, specificity, sensitivity, accuracy, precision, limit of detection (LOD) and limit of quantification (LOQ). The developed method was validated according to ICH guidelines for linearity, accuracy and precision, and specificity [21, 22].

Peak purity of all the drugs in HBSS buffer was assessed through the study of purity plots using PDA detector. Linearity of the method was evaluated from the standard curve of the detector response (peak area) against drug concentration. Calibration curve (n=3) with eight concentrations was plotted in the range of 5–100 μM. Peak areas of the drug versus concentration were plotted and found to be linear in the entire concentration range.

Accuracy and precision were determined with six replicates of quality control (QC) samples. QC samples were prepared in blank HBSS samples, at three concentrations of 10, 40, and 80 μM, following the same procedure as reported for calibration standards and using a different primary stock. Calibration curves were prepared thrice on the same day to assess the intra-day variation, and the inter-day variability was checked by constructing calibration curves on three consecutive days. Samples were analyzed at three different concentrations, in triplicate, within the calibration range (n=6). The results were expressed as percent relative standard deviation (% RSD) of concentration.

To determine the robustness of the developed method, experimental conditions were altered deliberately and the resolution between the drugs was recorded. The flow rate of the mobile phase was 1 ml/min. To study the effect of flow rate on the resolution, the flow rate was changed by 0.2 units from 0.8 to 1.2 ml per min. The effect of column temperature on resolution was studied at 35 and 45°C instead of 40°C. Also, the resolution of the drug was studied by performing the analyses on a different chromatographic system to establish the robustness of the method.

## Result and Discussion

### Method Development

Method development was initiated with aqueous phase and acetonitrile in 50:50 v/v proportions. Firstly, aqueous phase adjusted with buffers to various pH values, viz., pH 3.0 (phosphate buffer), 5.0 (acetate buffer) and 7.0 (phosphate buffer) were tried. However, acceptable results in terms of peak resolution could not be obtained. Thereafter, water adjusted to pH 3.0, 3.5, 4.0 and 5.0, with phosphoric acid in 50:50 proportion with acetonitrile was tried and a pH of 3.0 was chosen for further method development. The effect of pH was most prominent on FSD than on other drugs and at pH 5.0, FSD showed a doublet of equal intensity with peaks of the other drugs being unaffected.

Adjustment to pH 3.0 was tried using different acidifiers viz. trifluroacetic acid, hydrochloric acid, glacial acetic acid and phosphoric acid, but only the latter could separate all the drugs with good resolution and peak intensity. However, the resolution of peak was not optimal, with acetonitrile alone as the organic medium. Replacement of acetonitrile with methanol also could not improve the resolution. Thereafter, to improve the resolution and peak shape, mixtures of varying proportions of methanol and acetonitrile were tried, alongwith an aqueous phase adjusted to pH 3.0 with phosphoric acid. Thus, 50:50 v/v ratio of acetonitrile:methanol was finalized for further development. Increase in the concentration of either acetonitrile or methanol alone resulted in unresolved peaks and/or tailing, respectively. Concentration of the organic content (50:50 acetonitrile:methanol) in the mobile phase was varied from 20% to 50%, which led to selection of 40% v/v organic phase (50:50 acetonitrile:methanol), in combination with water pH 3.0 (60% v/v). Further optimization resulted in ratio of 58:42 v/v of water pH 3.0 and mixture of acetonitrile and methanol (50:50 v/v), respectively. However, application of a gradient method to further shorten the duration was unsuccessful, as it led to loss of resolution between the adjacent drug peaks.

### Stability of analytical solutions

Drug solutions of 100 μM (in triplicate) were kept in HBSS buffer for 24 h for bench-top stability study and analyzed using the same method. The results showed not less than 99 % of the drug remained in solution after 24 h (data not shown).

### Method Validation

#### Peak purity assessment

Peak purity evaluation was performed with the objective of obtaining supportive information during selection of appropriate analytical conditions for specific determination of permeability markers. Peaks corresponding to the drug showed positive value for the minimum peak purity index over the entire range of integrated peak indicating their purity.

#### Specificity and Sensitivity

**Fig. 1:**
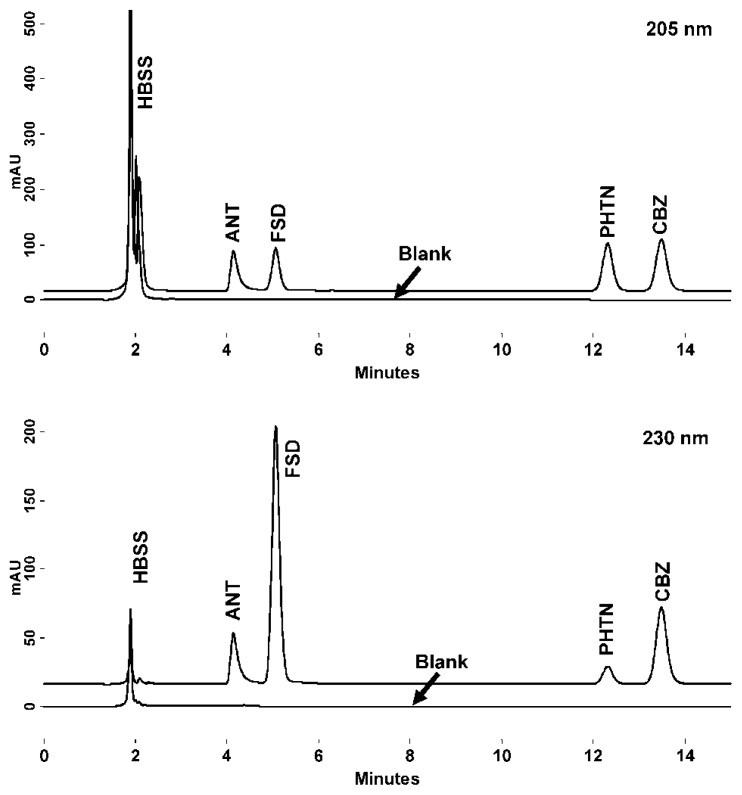
Chromatogram of ANT, CBZ, FSD and PHTN from mixture (100 μM) and blank HBSS buffer (lower) at 205 nm and 230 nm

Specificity of a method can be defined as absence of any interference at retention times of peaks of interest and is evaluated by observing the chromatograms of blank samples and calibration samples spiked with drug. Specificity is the ability of the analytical method to measure accurately and specifically the analyte of interest in the presence of other components that might be expected to be present in the sample matrix. The representative chromatograms of ANT, CBZ, FSD, and PHTN are presented as shown in Figure 1. Interfering peaks of any endogenous buffer are not observed near the retention times of these drugs. The retention times of ANT, FSD, PHTN, and CBZ were 4.1, 5.1, 12.3 and 13.5 min, respectively. The method specificity was assessed by comparing the chromatograms obtained from drugs alone, mixture of drug samples and through the peak purity curves.

The sensitivity of a method is represented by the values of limit of detection (LOD) and limit of quantification (LOQ). The lowest concentration of the drug detectable by the proposed method is termed as LOD, while LOQ is the minimum quantifiable concentration of the drug by the suggested method. The values of LOD and LOQ were computed by using the formula for detection limit as DL= 3 σ/S and for quantitation limit as QL=10 σ/S. The LOQ calculated using this formula was found to be 5.28 μM for ANT, 2.24 μM for CBZ, 0.58 μM for FSD and 2.04 μM for PHTN. The validation parameters including LOD and LOQ are shown in Table 2.

#### Linearity and Range

Table 3 shows the data for the determination of linearity and range for determination of ANT, CBZ, FSD and PHTN. As shown, the responses for the drugs were obtained to be strictly linear in the concentration range of 5–100 μg/ml. The standard curve had a reliable reproducibility over the concentrations of drugs across the calibration range.

#### Accuracy and Precision

Accuracy and precision of the method represents the repeatability and robustness of the analytical method and was determined by analyzing the QC samples. Good recoveries were obtained for each concentration used, confirming that the method was accurate, as shown in Table 4. Acceptable relative standard deviation (R.S.D.) limits indicated that the method is accurate and precise for the designated application.

#### Robustness

Resolution of the drugs in a mixture was found to be similar as in Figure 1, when studies were performed under deliberately altered conditions, indicating that the method had high robustness. Inter-day and intra-day calibration curves demonstrated the intermediate precision of the method and expressed as percent RSD for a statistically significant number of samples (n=6). The % RSD values in the regression lines prepared on the same day or different days were within the limits (Table 5).

### Application of method in determining Caco-2 permeability of markers

Permeability study of ANT, CBZ, FSD and PHTN (n ≥ 5) was carried out using Caco-2 cell monolayer grown on a polycarbonate filter for 21–25 days. The mean apparent permeability values (cm/s) of FSD, ANT, CBZ and PHTN were found to be 5.4 × 10 ^−6^, 106.9 × 10 ^−6^, 209.2 × 10 ^−6^ and 32.8 × 10 ^−6^, respectively, showing that FSD is a low permeability drug [23], and ANT [4], CBZ [24] and PHTN [25] belong to the category of high permeability drugs, as stated in the literature. The method is currently being routinely employed for analysis of markers for Caco-2 permeability studies.

## Conclusion

Assessment of *in-vitro* Caco-2 permeability requires simultaneous quantitation of high and low permeability markers to ascertain the suitability of the method. This study represents a simple, rapid and reliable validated RP-HPLC method for the quantification of ANT, CBZ, FSD and PHTN in presence of HBSS buffer with a relatively short run time. The developed method is highly specific, accurate and precise, making it suitable for the routine analysis of these permeability markers in Caco-2 permeability study.

## Figures and Tables

**Tab. 1:** Characteristics of permeability markers used

Drug	Structure	Molecular Formula	log P	pK_a_	BCS class
Antipyrine	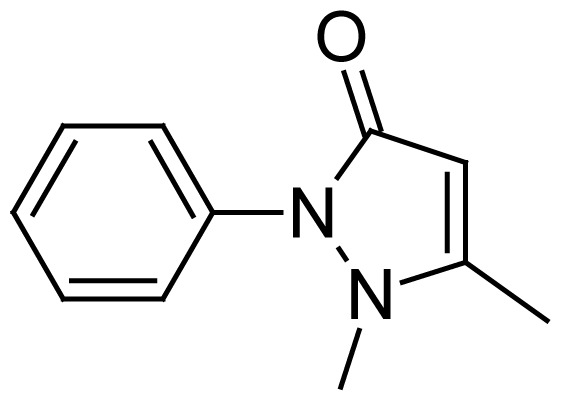	C_11_H_12_N_2_O (188.23 g/mol)	0.38	1.4	I (HP, HS)
Carbamazepine	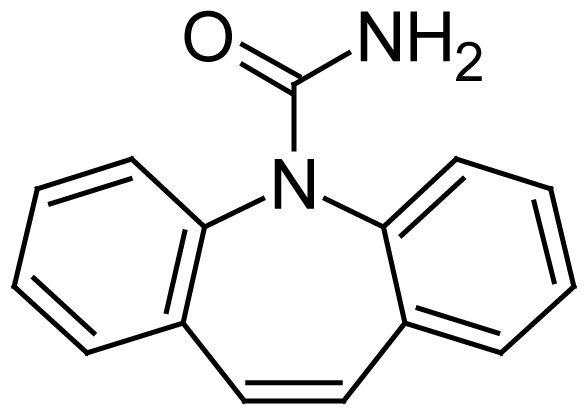	C_15_H_12_N_2_O (223.25 g/mol)	2.45	14	II (HP, LS)
Furosemide	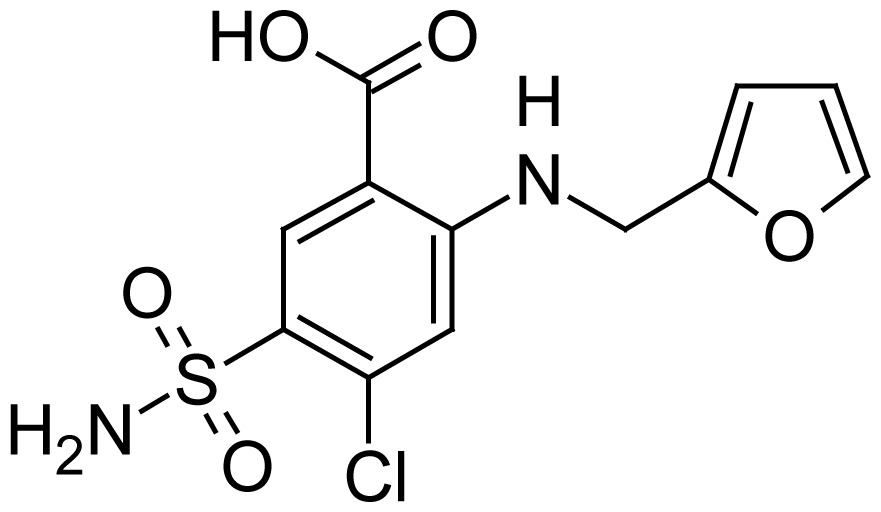	CH_12_Cl_11_N_2_O_5_S (330.74 g/mol)	1.4	3.9	IV (LP, LS)
Phenytoin	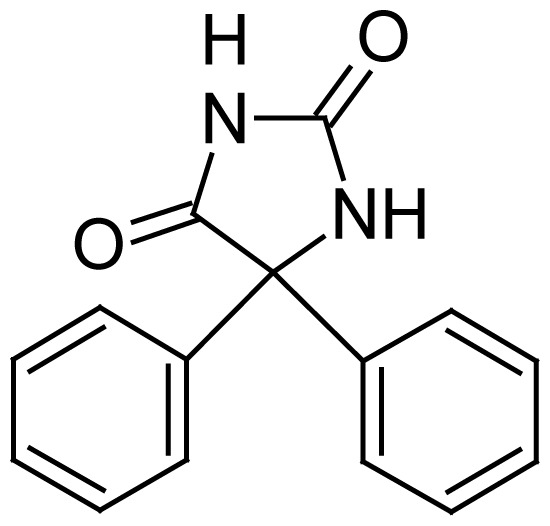	C_15_H_12_N_2_O_2_ (252.27 g/mol)	2.2	8.3	II (HP, LS)

HP…high permeability; LP…low permeability; HS…high solubility; LS…low solubility.

**Tab. 2:** Validation parameters of the HPLC method for ANT, CBZ, FSD and PHTN

Parameter	Value			
	
	ANT	CBZ	FSD	PHTN
Analytical wavelength	230 nm	205 nm	230 nm	205 nm
Range (μM)	5–100	5–100	5–100	5–100
Slope (Mean ±S.D; R.S.D.)	7668.8 ± 103.3; 1.3	16075.5 ± 39.3; 0.24	22085.1 ± 149.0; 0.67	13577.6 ± 18.19; 0.13
Intercept (Mean ± S.D.)	−5541.3 ± 172.8	−3746.8 ± 66.91	1426.1 ± 191.9	−3443.4 ± 386.1
Regression coefficient	0.999	1.000	0.999	1.000
LOD (μM)	3.28	0.67	0.13	0.73
LOQ (μM)	5.28	2.24	0.58	2.04

Each standard curve was generated in triplicate on 3 consecutive days, across the linearity range. Values are reported as mean±SD of three calibration curves.

**Tab. 3:** Data of linearity studies for ANT, CBZ, FSD, and PHTN

Conc. (μM)	Mean Peak Area ± S.D., R.S.D. (%)			

	ANT	CBZ	FSD	PHTN
5	33934.5 ± 130.8; 0.38	73873.3 ± 666.3; 0.91	106796.3 ± 1187.9; 1.11	61020.0 ± 400.2; 0.65
10	74048.5 ± 1948.1; 2.63	159297.5 ± 4349.4; 2.73	220881.0 ± 5107.4; 2.31	132452.5 ± 1699.2; 1.28
20	147243.0 ± 861.25; 0.58	314557.3 ± 1862.6; 0.59	443556.3 ± 1103.3; 0.25	264798.0 ± 625.1; 0.24
30	225102.5 ± 1723.2; 0.76	483318.7 ± 2727.5; 0.56	677090.7 ± 1529.9; 0.22	407881.0 ± 1223.3; 0.29
40	297507.0 ± 381.8; 0.13	644809.0 ± 1289.9; 0.20	900838.7 ± 2264.2; 0.25	544944.5 ± 1320.2; 0.24
60	459703.0 ± 445.9; 0.09	966179.0 ± 5876.6; 0.61	1341402.0 ± 1512.6; 0.11	814444.0 ± 1694.2; 0.21
80	614150.3 ± 1137.8; 0.18	1274946.0 ± 3176.9; 0.25	1773965.0 ± 2945.5; 0.17	1076016.0 ± 2026.6; 0.19
100	776083.0 ± 76.4; 0.01	1612014.0 ± 6691.5; 0.41	2223262.0 ± 4658.7; 0.21	1358006.0 ± 653.4; 0.05
Equ.	y = 7765.3x − 5694	y = 16113x − 3773	y = 22256x + 1535.3	y = 13596x − 3881
(r^2^)	0.999	1.000	0.999	1.000

**Tab. 4:** Result of specificity study

Conc. (μM)	ANT		FSD	

	Conc. ± S.D., R.S.D. (%)	%R	Conc. ± S.D., R.S.D. (%)	%R
10	10.145 ± 0.137; 1.35	101.45	9.955 ± 0.095; 0.95	99.55
40	39.805 ± 0.367; 0.92	99.51	40.186 ± 0.124; 0.31	100.46
80	80.192 ± 0.444; 0.55	100.24	79.960 ± 0.137; 0.17	99.95

**Conc. (μM)**	**PHTN**		**CBZ**	

	**Conc. ± S.D., R.S.D. (%)**	**%R**	**Conc. ± S.D., R.S.D. (%)**	**%R**

10	9.929 ± 0.137; 1.38	99.29	10.123 ± 0.172; 1.70	101.23

40	40.137 ± 0.136; 0.34	100.34	40.209 ± 0.136; 0.34	100.52

80	79.826 ± 0.266; 0.33	99.78	79.833 ± 0.223; 0.28	99.79

%R… % recovery.

**Tab. 5:** Precision studies (n = 6)

Spiked Conc. (μM)	Measured Concentration ± S.D., R.S.D. (%)			

	ANT	FSD	PHTN	CBZ
**Inter-day precision**				

10	10.247 ± 0.183; 1.78	10.041 ± 0.087; 0.86	9.936 ± 0.045; 0.45	10.114 ± 0.125; 1.23
40	40.025 ± 0.645; 1.61	40.589 ± 0.248; 0.61	40.318 ± 0.286; 0.711	40.252 ± 0.254; 0.63
80	79.787 ± 0.916; 1.14	80.007 ± 0.389; 0.48	79.706 ± 0.402; 0.50	79.795 ± 0.552; 0.69

**Intra-day precision**				

10	10.374 ± 0.068; 0.66	10.015 ± 0.03; 0.31	9.965 ± 0.026; 0.26	10.183 ± 0.115; 1.13
40	39.610 ± 0.189; 0.47	40.748 ± 0.131; 0.32	40.387 ± 0.121; 0.29	40.217 ± 0.152; 0.37
80	79.803 ± 0.326; 0.41	80.256 ± 0.267; 0.33	79.647 ± 0.128; 0.16	79.661 ± 0.126; 0.15
